# Trading with richer and poorer countries: trade integration and regional inequality in Greece

**DOI:** 10.1007/s00168-021-01062-1

**Published:** 2021-06-07

**Authors:** Andrés Rodríguez-Pose, Alexandra Sotiriou

**Affiliations:** Cañada Blanch Centre and Department of Geography and Environment, London School of Economics, Houghton Street, London, WC2A 2AE UK

**Keywords:** Trade, Regional inequality, Economic growth, Greece, F13, O24, R11, R12

## Abstract

This paper examines the link between increased trade and regional GDP growth across the regional income distribution in Greece during the post-EMU period (2000–2013). By means of quantile regression techniques, panel fixed effects and system generalized method of moments (GMM), we disentangle the effects of EU trade—trading with generally richer countries—versus global trade—in the case of Greece, mostly trading with poorer countries—at several points of the regional income distribution to identify differences in trade elasticities. The analysis finds that the impact of EU trade is highly heterogeneous and mainly affects negatively the economy of the richer regions in Greece. In contrast, the effects of EU trade display insignificant results for the lower-income regions, attributed to the absence of direct substitution effects.

## Introduction

Trade integration is normally expected to unravel a multitude of benefits in terms of growth-inducing factors, such as larger market access, productivity, and knowledge transfer gains. However, it may also pose a serious of threats, such as substitution effects for the domestic competing industries and to those vulnerable regions more exposed to the trade integration dynamics (Petrakos et al. 2012; Autor et al. 2013). Increased trade is expected to produce large macro-economic benefits, but not all territories are likely to benefit in the same way. While some regions within a country may reap the lion’s share of these benefits, others may lose out both in terms of economic performance and employment, triggering negative social and political reactions.

This paper aims to contribute to our understanding of the geographically uneven effects of increased trade in a peripheral EU economy, following accession to the Eurozone. Given the observed spatial imbalances in both the evolution of trade and the growth trajectories of EU regions (Cappelen et al. 2003; Camagni and Capello 2009), our analysis aims to uncover the mechanisms behind the relatively poor performance of Greek regions in a context of deepening European and global trade. As EU integration intensified, the great majority of Greek regions stagnated. The lack of economic dynamism of regions in Greece has attracted the attention of international scholars seeking to reveal the mechanisms and shed light on its causes (e.g., Rodríguez-Pose et al. 2012; Petrakos et al. 2012; Petrakos and Psycharis 2016). Among the salient factors identified are competition pressures related to the widening and deepening of the EU integration process (Petrakos et al. 2012); the inability of the Public Investment Programmes in promoting regional convergence and narrowing the development gap among Greek prefectures (Rodríguez-Pose et al. 2012); the specialization of Greek regions in export-declining sectors that experience labor productivity losses (Kallioras et al. 2016); and the peripherality of the Greek economy compared to the EU core. Studies also stress the institutional weaknesses and the limited effectiveness of the adjustment programmes (Kotios et al. 2017), the uneven growth returns of the EU Structural Funds programmes (Sotiriou and Tsiapa 2015), and the severe impact of the financial crisis (Petrakos and Psycharis 2016).

The case of Greece is important on account of a number of prominent reasons. Greece became part of the Eurozone despite lower levels of economic competitiveness than other Eurozone members. It is characterized by a weaker industrial base in terms of economies of scale and regional inequalities are pronounced. The examination of the Greek case also presents a setting for external validity to other peripheral economies, while the phenomenally deep recession that affected Greece post-2009 has to be partly seen in light of the previous failures of Greek regions in securing higher competitiveness and sustainable growth.

Greece’s accession to the Eurozone (2001) led to a rapid deepening of trade integration. The effect of the common currency reduced overall transaction costs, including borrowing costs and facilitated the surge of imports. Although trade liberalization (i.e., inclusion into the EU common market) started in the late 1980s, it was after the mid-1990s that imports from the EU more than doubled, while exports followed a less pronounced upward trend. The combination of intensified trade with the EU, the uneven distribution of economic activity in the Greek territory due to its concentration in two main urban centers (Psycharis et al. 2014), as well as the heterogeneity of Greek regions with respect to their structural characteristics, provide an interesting setting to empirically explore the regional response to the deepening of trade in a peripheral economy.

Empirical research on regional disparities has identified a mosaic of determinants explaining uneven growth trajectories. These include geography, initial levels of income, human capital endowments, the quality of infrastructure as well as accessibility and institutional deficiencies (Midelfart-Knarvik and Overman 2002; Crescenzi and Rodríguez-Pose 2012; Rodríguez-Pose et al. 2012; Monastiriotis et al. 2017). The extent to which trade acts as a causal mechanism of uneven growth at the sub-national (regional) level is much less understood and the evidence appears inconclusive (Paluzie 2001; Petrakos et al. 2012; Kallioras and Pinna 2015).  Overall, different types of trade affect regions at different levels of development differently (Rodríguez-Pose 2012).

This paper analyses the differential economic effects of rapid increases in trade with different types of countries for the case of Greece. It pitches EU trade integration—generally with countries wealthier than Greece—vis-à-vis trade with the rest of the world, which—with the exception of energy-related trade—involves mostly countries with a lower level of development and lower labor costs than Greece. These different types of trade integration are expected to have heterogeneous competition effects and thus produce a diverse set of growth-inducing and substitution effects across the regional income distribution. We thus contribute to the existing debate by explicitly testing the hypothesis that the economic development of a region, and specifically its position in the regional income distribution, will influence the nature and intensity of the trade effect and that this effect will depend on the type of trade being undertaken.

The heterogeneous trade integration effects will be evaluated by means of quantile regression techniques. Such techniques allow testing whether the impact of increased trade differs according to the income level of a region. We thus test the hypothesis that more advanced regions will be more affected by EU trade, due to their relatively similar sectoral structure to the composition of EU trade flows (compared to non-EU trade), which, in turn, intensifies import competition pressures. In other words, a more advanced region will produce (and export) a more similar bundle of goods and products to those of its core EU trade partners, compared with a poorer, agricultural, more ‘sheltered’,^1^ and peripheral region. This renders the competition from the EU fiercer for the advanced region compared to the poorer region.

Consequently, substitution effects from EU trade (especially intra-industry EU trade) will affect richer regions and will impact poorer regions less, as they do not directly compete with the more advanced EU imports. The methodological novelty of the analysis is to detect the heterogeneity of the trade-growth nexus across the regional income distribution by decomposing the effects of two different types of trade integration.

Although quantile regression techniques have received attention in cross-country growth studies (e.g., Crespo-Cuaresma et al. 2011), the regional dimension remains underexplored (Costa-i-Font and Rodríguez-Oreggia 2005). Additionally, the parameter heterogeneity of a number of growth determinants across regions provides fruitful insights for policy recommendations.

The remainder of the paper is organized as follows. In Sect. 2 the theoretical framework and review of the empirical evidence are presented. Section 3 provides a descriptive analysis of EU trade and the regional growth performance in Greece. Section 4 is devoted to methodology and empirical results, while in Sect. 5 we conclude and discuss some policy implications.

## The geographically uneven impact of trade

### Spatial implications of trade on growth

The theories on the intra-national spatial distribution of the benefits stemming from trade integration stem primarily from the new economic geography (NEG) theory (Krugman and Livas Elizondo 1996). The corresponding models are mostly concerned with the question of “whether increasing cross-border integration leads to a greater intra-national concentration of manufacturing activity, thereby increasing regional inequality” (Rodríguez-Pose 2012: 112). According to these theories, trade can give rise to core-periphery patterns, which are the outcome of the interplay between centripetal and centrifugal forces. These forces depend on a set of assumptions pertaining to transport costs and the mobility of (agricultural and manufacturing) labor (Paluzie 2001). As such, in the theoretical literature, the trade-growth relations at the regional level are framed only implicitly as a response to the relocation of economic activity across space.

From a theoretical perspective, the geographical implications of trade start with the neoclassical trade theory. Under a Heckscher–Ohlin framework, trade is based on the comparative advantage principle, with theoretical models that focus on the relationship between international trade and the distribution of income across countries trading with each other. From this perspective, free trade would benefit any factor used exclusively in the production of exportable commodities and harm those factors used in the import-competing sector. The key implication of the model is that the set of industries produced by a country is a function of its relative endowments. In an open world trading system, relatively capital- and skill-abundant countries, like the core EU countries, will manufacture a more capital- and skill-intensive mix of products than relatively labor-abundant countries, like southern EU countries, including Greece.

The neoclassical theory predicts that trade in goods between regions with different endowments of production inputs (e.g., skilled and unskilled labor, capital) equalizes the relative wages and rates of return to capital in each country/region. For example in places with an abundance of unskilled labor relative to skilled labor, such as the less developed regions of Greece, unskilled wages would be low in the absence of trade. In the presence of trade, Greece will specialize in the product or products using the abundant (unskilled) factor. This will lead to a rise in wages associated with the unskilled-intensive product, due to the increase in the demand of the exported product. The geographical consequence of this will be convergence across regions (Nello 2012). However, this assumes no factor mobility and diminishing returns to factor inputs. In reality the bundle of products that a country/region exports and imports is more complex and comprises of both skilled and unskilled-intensive products—as in the case of Greece. Hence, the spatial outcomes become more complicated and less predictable in terms of the effects of import competition across the entire skill- and capital-intensive spectrum. From this perspective, the neoclassical trade theory is unlikely to explain the surge of intra-industry trade between countries with different endowments and income levels and the spatial growth impact at the regional level. This is particularly the case in a country such as Greece, with large internal industrial and economic contrasts.

The New Trade Theory (NTT), the New Economic Geography (NEG), and the endogenous growth theory also have considerable implications for the geography of trade. The NTT explains the surge of intra-industry trade as a result of increasing returns to scale and product differentiation. NEG and, in part, the endogenous growth theory, offer the theoretical foundations for explaining the growth returns of trade in large agglomerations. One of the main predictions of NEG supported by empirical evidence is that the reduction in trade costs—in combination with increasing returns to scale—is expected to increase the growth returns from trade in large urban agglomerations (e.g., Ottaviano et al. 2002), thus producing uneven growth patterns. The gradual reduction in transport costs worldwide and the reduction in transaction costs from the creation of the Eurozone played a catalyst role in the deepening of EU trade integration. The outcome has been within-country concentration trends in manufacturing and services (Brülhart and Traeger 2005).

The question to be answered is “if long term growth is driven by the endogenous accumulation of experience through learning-by-doing, then trade between regions can lead one region to specialize in industries in which it has a comparative advantage (e.g., traditional economic activities), but for which the opportunities to learn are relatively small, so that the growth rate in that region may be lower precisely because of trade integration” (Martin 2001: 6). With initial conditions reflecting the specialization of core areas on higher value-added industries, trade and, in particular, the reduction of transaction costs are expected to enhance the spatial concentration of increasing returns to scale activities in the core. By contrast, the periphery will specialize in industries with constant returns to scale (Martin 2001).

Acting via knowledge transmission channels, trade may amplify core-periphery patterns by benefiting disproportionately core areas with high concentrations of specialized labor and other prior advantages of higher levels of R&D expenditure, physical and human capital, and better institutions. Clustering is expected to occur due to the prevalence of agglomeration forces, such as firm-level economies of scale and external economies of scale. This, in combination with highly specialized labor turnover and reduced trade (transport and transaction) costs, creates virtuous cycles of growth in core regions (Nello 2012). As per the NEG, the surge of trade will thus benefit core regions to a far larger extent than regions in the periphery that lack the favorable geography and initial conditions to compete successfully in an integrated market.

The endogenous growth theory predicts that trade generates positive externalities and spillover effects, which facilitate the transition and dissemination of technological progress, knowledge, and ideas (Fine 2000). However, this may not be the case when trading counterparts exhibit considerable differences in terms of endowments and level of technology, while lagging economies may find it difficult to grasp the dynamic effects of trade also attributable to the lack of absorptive capacities (Devereux and Lapham 1994). To this end, as Martin (2001: 2) argues “because neither policy-makers, nor economists are ready to give up the gains from trade integration, a natural implication is to employ public policies to counteract the possibility of increased regional inequalities which are viewed as unacceptable on distributional and political grounds”. The latter signifies that uneven or even negative net effects from deeper EU trade integration may affect some regions—and, in particular, less developed regions—, which urges regional policy intervention.

The main theoretical model on the spatial effects of trade was constructed by Krugman and Livas Elizondo (1996). They have since been challenged by other scholars, such as Paluzie (2001). Using the example of Mexico, Krugman and Livas Elizondo (1996) show that trade liberalization brought a de-concentration of industrial activity and shifted it away from Mexico City to the northern states (due to proximity to the US border and the centrifugal forces of congestion). They explain that the economic landscape changed rapidly after the country opened up to trade and economic activity evened out as inputs could be sourced from abroad and output was exported. A dispersal of manufacturing activity ensued, reducing regional disparities.^2^

This view was later challenged by Paluzie (2001). Departing from similar assumptions,^3^ she constructs a model to explain the evolution of regional inequalities in Spain. She finds that the opening up of a closed economy brought further regional polarization that coincided with the interruption of the convergence process in the EU and especially in Spain (in the 1980s). This model adheres more to the basic Krugman core-periphery model in which labor mobility plays a key role in reinforcing the unequal geography of trade and regional polarization, which comes as a result of trade liberalization (Paluzie 2001). For the study of the regional growth-trade nexus, such theoretical framework is limited, as the existing theoretical models in the NEG literature use the fluctuation in transport costs to determine the location of economic activity. This, in turn drives the evolution of regional disparities. Much less is spelled out in relation to the actual benefits and costs of increased trade, conditional on the wealth of regions. The existing empirical literature has relied mostly on broad typologies of rural versus urban areas or poor versus rich countries (e.g., Rodríguez Pose and Gill 2006; Rodríguez-Pose 2012; Krugman and Livas Elizondo 1996).

In the specific case of Greece, considering that the bulk of trade between Greece and the EU is concentrated in the manufacturing sector (Petrakos et al. 2012), the above models offer insight for the study of regional growth trajectories. The surge of trade for an EU peripheral small country, such as Greece, has led to high trade deficits, due to the inability of the economy to compete with the economies of scale of northern EU industries. This has resulted in import competition and substitution effects that harm the incumbent industry in manufacturing hubs (e.g., Petrakos et al. 2012), affecting disproportionally the growth trajectories of more advanced regions.

### Trading with richer and poorer countries and its spatial consequences

To what extent does trading with countries at different points in the wealth and technological scale affect the economic performance of regions within a country? Although this topic has received relatively limited attention, research is increasingly showing that trade leads to uneven growth patterns at the subnational level, suggesting heterogeneous responses to increases in trade exposure (Rodríguez-Pose 2012; Autor et al. 2013; Petrakos and Psycharis 2016). There is a growing consensus that trading with more advanced economies often benefits the core areas within countries rather than peripheries. López-Bazo et al. (1999), for example, found that deeper EU trade integration resulted in poorer regions suffering “higher disequilibrium in their labour markets” (López-Bazo et al. 1999: 366), resulting in negative effects on growth and convergence.

Greece followed this trend. The opening of the Greek economy to trade with the more advanced countries of the EU—Greece was the poorest member at the time of joining in 1981—coincided with a strengthening of the concentration of economic activity in the core urban cities and manufacturing hubs. The result was growing regional inequalities, in terms both of the location of economic activity and the growth prospects of regions. Higher value-added manufacturing production became more concentrated in the upper quantiles of the regional income distribution leading to rising regional disparities (Caraveli and Tsionas 2012).

Moreover, changes in trade patterns seem to affect the evolution of regional inequality in poorer countries to a far greater extent than in richer ones. In the case of less developed countries, Rodríguez-Pose and Ezcurra (2014) find a positive link between trade and spatial inequality in 22 developing countries over the period 1990–2006. They conclude that a greater degree of trade openness reduces the GDP per capita of poorer regions and increases that of richer regions, thereby creating winners and losers that coincide with rich and poor regions respectively.

But increases in trade do not necessarily always benefit the more developed regions of a country at the expense of the poorer ones. When the timing of the opening to trade is considered, the territorial impact of trade can be quite different. As Kallioras and Petrakos (2010) have shown for the case of the post-2004 member states, regions initially more exposed to trade competition with the rest of the EU suffered more in terms of industrial employment destruction. Hence, more advanced and industrialized regions—but with weak industrial structures—are bound to suffer significant employment losses following increases in trade and economic integration with more advanced economies. Specifically, the impact of EU integration in the post-2004 member states was fiercer for the more industrially advanced regions—i.e., the regions that, at the time, produced the greatest part of the national industrial gross domestic product (Kallioras and Petrakos 2010). These regions, however, also enjoyed the best conditions—from agglomeration economies, better human capital endowments, or more innovation capacity—to combat this decline. Although the relationship between the level of development of a region and the exposure to certain trade partners may go both ways, suggesting that more economically advanced regions will trade with richer countries and vice versa, our analysis by taking into account the heterogeneity of the income level of the trade partners in part addresses the differing impact on regional growth.

Very often and despite the generalized view that trade is beneficial for all parties engaged, trade integration can be traumatic. Studies on the first decade of Greece’s participation in the EU have stresses that, despite a long transition period following EU accession, the Greek economy recorded no export gains and saw a marked decline in competitiveness attributed to high levels of import penetration (Arghyrou and Bazina 2003). At the sub-national level, empirical evidence on Greece’s EU integration experience, revealed the inability of Greek regions to compete (successfully) with their more advanced counterparts in capital-intensive manufacturing and knowledge-intensive economic activities (Petrakos et al. 2012). The export performance of most sectors in Greek regions remained weak, highlighting the role played by competition from imports in producing negative employment (growth) effects (Fotopoulos et al. 2010). Regional idiosyncratic features drove employment growth patterns, with domestic demand, stronger in the largest agglomerations, standing out as the critical driver of regional employment growth (Fotopoulos et al. 2010).

Hence, while trade integration with more advanced countries can have positive and negative economic impacts for more and less developed regions within a country engaging in trade, in the case of Greece, we draw on the insights of Petrakos et al. (2012) expecting that, as richer regions host higher shares of industrial activity (Caraveli and Tsionas 2012), trade integration increased competition pressures there to a far greater extent than in more 'sheltered' or poorer regions. Trade with mainly less developed countries in the rest of the world—focused mainly on low level manufacturing and agricultural produce—has posed, by contrast, less of a threat for the industry of core regions, while imperilling those in less developed areas of Greece. The industrial sector is considered the main diffusion channel of the integration dynamics in a spatio-structural context (Amiti 1998), due to “the displaceable character of its activities, the tradable character of its products and the linkages it retains with the other sectors of production” (Petrakos et al. 2012: 347).

## Trade and regional inequality in Greece: Facts and figures

**Fig. 1:**
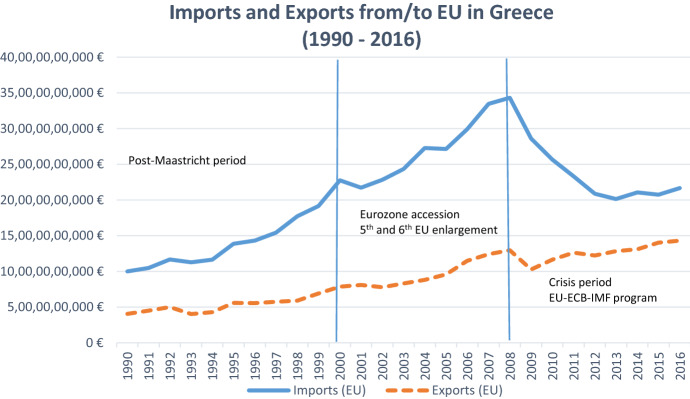
Evolution of Greece’s Trade with the EU

Source: own elaboration using data from Eurostat (*International trade database*).

The EU has traditionally been the most significant trade partner for Greece, Fig. 1 sketches the evolution of EU imports into Greece as well as Greek exports to the EU. From 2000 onwards there was a sharp climb of EU imports, which coincided with the accession into the Eurozone. The high absorption of EU imports was fuelled by the lower transaction costs of the common currency union and the large reduction in borrowing costs (interest rates fell from 25 to 5%) for both corporations and consumers. In the same period, there was also an upward trend in exports. However, the gap between imports and exports widened significantly after accession up until the crisis erupted in late 2008.

**Fig. 2:**
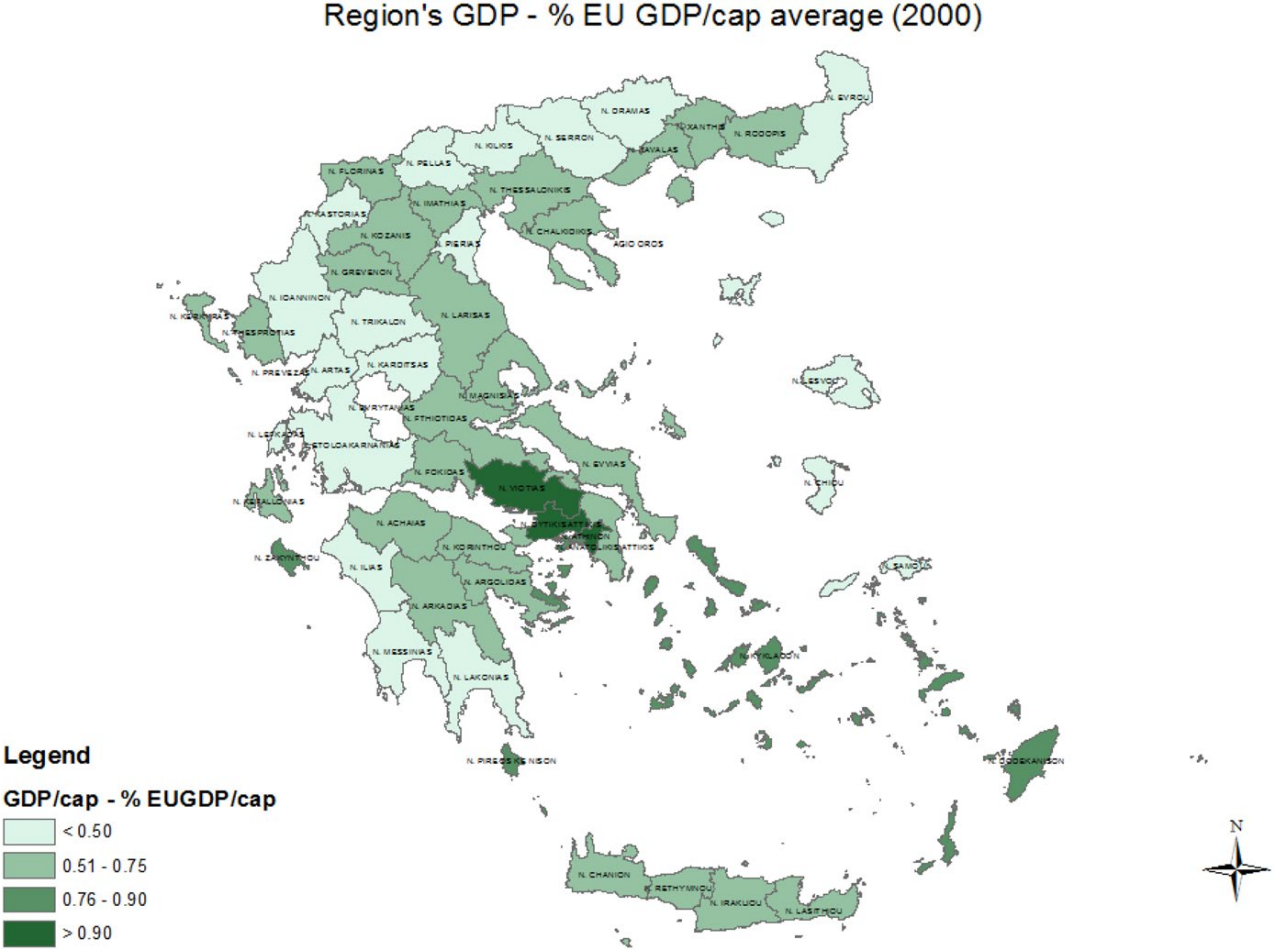
Map of Region’s GDP as a percentage of the EU average (2000). Source: own elaboration using data from EUROSTAT

**Fig. 3:**
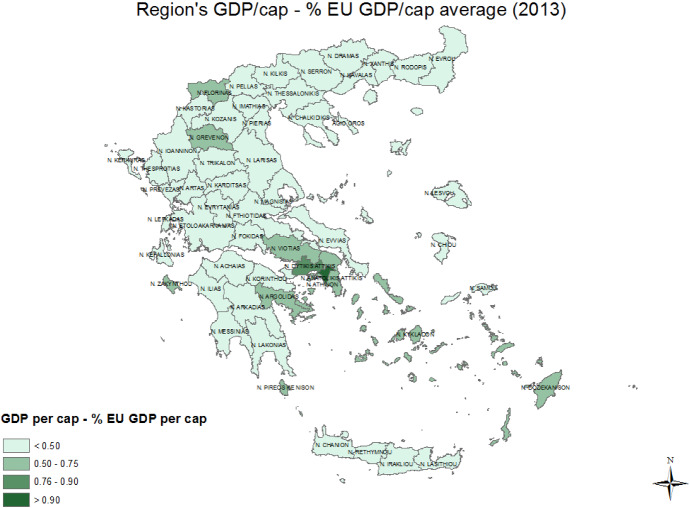
Map of Region’s GDP as a percentage of the EU average (2013). Source: own elaboration using data from EUROSTAT

Greece’s largest trade deficit expanded significantly in the 2002–2008 period, following Eurozone accession. This increase in the trade deficit happened in an economy with already stark regional inequalities. Figures 2 and 3 display the spatial heterogeneity of regional GDP as a percentage of the EU average for the years 2000 and 2013 respectively. As can be observed, with the exception of Attica and some of the islands, most Greek regions remained well below the *less developed* status of regions in the EU (75% of EU average) in 2000. The crisis meant that Greece went in economic reverse gear and by 2013 the vast majority of regions did not manage to surpass 50% of the EU average.

**Fig. 4:**
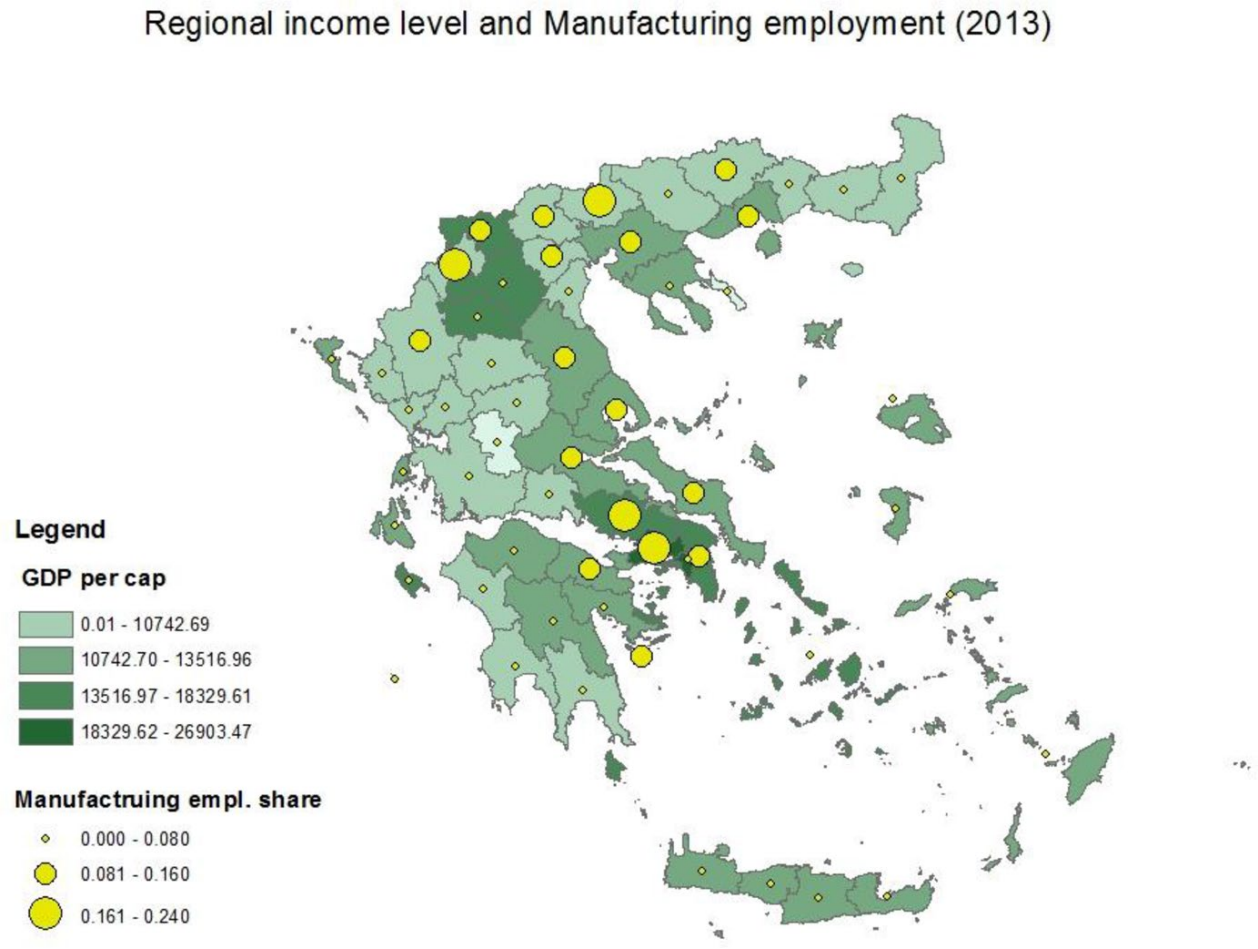
Map of Economic development and MNF share at NUTS III (2013). Source: own elaboration using data from EUROSTAT

Figure 4 offers a snapshot of the spatial distribution of the economic development of Greek regions (in terms of GDP per cap.) and the share of manufacturing employment by region. Darker colors correspond to higher levels of economic development and larger circles represent higher shares of manufacturing. The majority of the economically advanced regions (located on the Athens-Thessaloniki axis) host high shares of manufacturing, while the poorer regions in the north (bordering the Balkan region) specialize in lower value-added and more labor- and resource-intensive manufacturing. This pattern underlies the hypothesis that more advanced regions, which host higher value-added manufacturing, compete with EU imports to a greater extent than poorer regions. The relatively advanced regions in the southern Peloponnese and Crete with lower shares of manufacturing rely mainly on high value-added agricultural production or tourism.

**Fig. 5:**
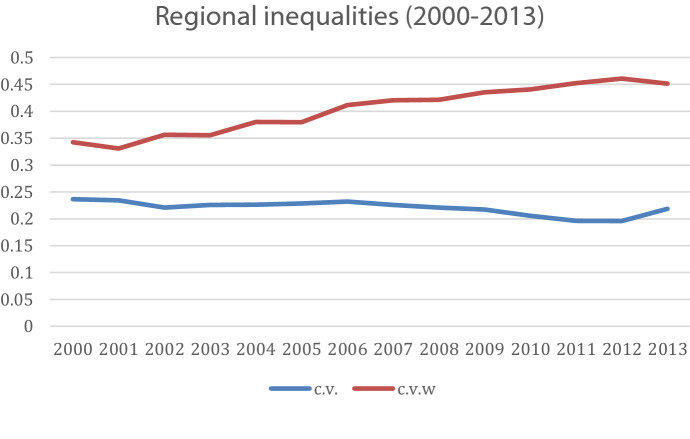
Evolution of regional inequalities. Source: own elaboration using data from El.STAT

Figure 5 illustrates the evolution of regional disparities based on the calculation of both the coefficient of variation (c.v.) of GDP per capita as well as the population-weighted coefficient of variation (c.v.w.), following Petrakos and Psycharis (2016). As depicted in the figure, the un-weighted coefficient of variation (c.v.) shows a stable and perhaps declining trend of regional inequalities that could be attributed to the underperformance of the more advanced regions. This trend is in line with the hypothesis on the more pronounced negative effects of increased EU trade for regions in the upper quantiles of regional income distribution. This may be a consequence of the contraction of the manufacturing sector, which has traditionally been concentrated in the mid-income and richer regions. Such contraction brings the more affluent regions closer to the regional income average, thus reducing regional disparities. However, the weighted C.V. follows a slight upward trend indicating a widening of regional inequalities, linked to “the dominant position that Athens has in the Greek economy and its relatively better performance than the rest of the regions which maintain the previous trends of divergence” (Petrakos and Psycharis 2016: 142).

## Methodology and description of data

The econometric analysis is based on a production-function specification, where regional output (Y) is modelled as a function of two main factors of production (capital and labor). Capital (Κ) is proxied by the expenditures of the public investment programme commonly used in the regional growth literature. Labor (L) is proxied by population (normalized on regional area size), which also accounts for size and urbanization. For the assessment of the impact of EU trade integration on regional GDP growth, the equation takes the following form:

1 $${\text{Log}} (Y_{r,t } ) = \beta_{1} TII_{r,t - 1} + \mathop \sum \limits_{k = 1}^{\nu } \beta_{k} X_{r,t - 1} + \delta_{t} + \varphi_{r} + \varepsilon_{r,t}$$

The model is extended with the addition of a set of controls *X*, which is a vector of *k* region-specific variables (growth determinants). The dependent variable is the natural logarithm of real GDP per capita in region *r,* at year *t;* region and year fixed effects (${\varphi }_{r} \mathrm{and }{\delta }_{t })$ are included in all models to capture idiosyncratic time-invariant differences in growth rates across regions and national business cycles, respectively. The model is equivalent to a growth model, as it is a panel fixed effects model with log GDP per cap as the dependent variable. The analysis covers the period corresponding to EMU accession—from 2000 to 2013—and is estimated at the NUTS III level (50 regions/prefectures). Prefectures in Greece have traditionally been the key spatial level for regional development policy (Rodríguez-Pose et al. 2012) and represent the most disaggregated administrative unit for which trade-related data are available. The empirical analysis is based on a balanced panel evaluated by means of a fixed effects regression and the use of quantile regression techniques. This is followed by a system-GMM estimation, with the aim of checking the robustness of the results, while simultaneously addressing issues of causality. Quantile regressions in the EU regional context have been previously used by Crespo-Cuaresma et al. (2011), indicating that growth determinants differ across quantiles.

The key regressor, TII, takes the form of two different types of trade integration: (a) trade integration with the EU, *EUT*, which represents trade with a group of countries that is, on the whole, more developed than Greece; and (b) trade with the rest of the world, *ROWT*, that, in the case of Greece, is to a large extent conducted with countries with a lower level of development than Greece. The construction of both indices is explained in detail below.

Following Petrakos and Psycharis (2016) and Petrakos et al. (2015), the main regressor for trade with more developed countries is the EU trade integration index (*EUT*), which is measured according to the following formula:

2 $$EUT_{r,t} = \frac{{M_{r,t}^{EU} + X_{r,t}^{EU} }}{{M_{r,t}^{W} + X_{r,t}^{W} }}$$

The index represents the ratio of imports and exports from/to the EU in region *r* and year *t* over the total imports and exports of region *r* in year *t*. This is an indicator of EU trade intensity. The variable is measured using the actual annual regional trade flows at the NUTS III level provided by the National Statistical Office of Greece (ELSTAT). We use actual trade flows at the region (*nomos*) level and not proxies based on location quotients, which was the method used in previous research. All variables and their sources are described in detail in the appendix (Table 6).

We factor in the level of *openness* of the region by including the trade openness index (*Openness*), which captures overall trade. The index follows the traditional definition of openness used widely in the literature: the ratio of overall trade (imports and exports) of the region over the region’s GDP (Frankel and Romer 1999; Frankel and Rose 2002; Ezcurra and Rodríguez-Pose 2014).

3 $$Open_{r,t } = \frac{{Trade_{r,t} }}{{GDP_{r,t} }}$$

We further test the effects of trade with generally less developed countries, by means of a rest of the world (*ROWT*) trade index. The majority of Greek trade with non-EU countries takes place with Turkey, non-EU Balkan countries, and countries in Asia and North Africa. Hence, trade integration with these markets is likely to produce smaller substitution effects, while the export capacities of Greek regions in less competitive non-EU markets are expected to be higher. The index is measured following the same formula as the previous index:

4 $$ROWT_{r,t} = \frac{{M_{r,t}^{nonEU} + X_{r,t}^{nonEU} }}{{M_{r,t}^{W} + X_{r,t}^{W} }}$$

*ROWT* is measured as the ratio of trade with non-EU countries over total trade by region and by year and captures changes in the trade intensity with countries outside the EU.

All variables are included with 1-year lags in order to capture potential delays of the impact of the main regressors and of the controls on regional development. The introduction of a lag also partly mitigates reverse causality concerns.

The vector of controls contains the following variables. First, the logarithm of population density (*log PopDen*). This variable has traditionally been included in standard economic development models and is a proxy of agglomeration economies and market size. However, the positive effect on growth is not always confirmed by the empirical literature. As noted by de Groot et al. (2011) and empirically tested by Psycharis et al. (2014), large agglomerations are more exposed to economic downturns and therefore often experience higher negative effects in periods of economic crisis than less developed areas. In Greece, regions hosting large cities or specializing in manufacturing (like Central Macedonia and part of Continental Greece, Thrace, and Thessaly) were hit harder due to “the difficulties of most industries in maintaining production in the face of reduced demand, severely cut bank credit, imported supplies and export guarantees” (Petrakos and Psycharis 2016: 143).

Second, public investment, using data from the Greek public investment programme (PIP). This programme is the main regional development tool for incorporating EU Structural Funds in Greece. The investment is measured as the per capita expenditure in region *r* in year *t (log PIP*). The programme has the aim of promoting convergence towards the standards of living of the EU and to reduce domestic regional asymmetries. However, the efficacy of public investment in Greece has frequently been questioned (e.g., Rodríguez-Pose et al. 2012; Monastiriotis and Psycharis 2014). The programme has been found not to be territorially progressive enough and to frequently fall prey to corruption, thus limiting its impact on growth (Rodríguez-Pose et al. 2012).

A third control variable is the level of connectivity (*accessibility*) of a region. This variable is measured as the inverse time-distance weighted population using road network data in the EU. The distance decay function is a fairly steep exponential function that approaches zero after four hours of travel. The connectivity of a region is expected to be positively related to growth, as it captures the growth dynamics from increased market potential. Finally, we also include the share of the public sector in the regional economy, measured as the ratio of GVA produced in the public sector over the region’s total GDP, following Petrakos and Psycharis (2016).

The aforementioned control variables are included both to improve the fit of the model and to test the robustness of the main regressors to the inclusion of alternative growth determinants. The analysis follows the use of quantile techniques, which provide insights in terms of the heterogeneity of the main regressors and the growth determinants across the regional income distribution.

## Empirical results

In the following section we compare the results of panel fixed effects models with quantile regression techniques to first assess the impact of increased trade intensity, overall openness and trade with EU and non-EU markets on the growth performance of the regions. Second, we identify the heterogeneous effects of the various types of trade integration across the regional income distribution. The hypotheses to be tested are the following:

**H**_**1**_**:** The geographical impact of trade strongly depends on where the trade is originated (in this case EU- versus ROW-trade), as trade origin captures the composition of trade flows.

**H**_**2**_**:** The level of development of a region—proxied by its income per capita—determines the heterogeneous impact of the two different types of trade on growth.

We expect that, to the extent that the production structure and sectoral profile of the better-off Greek regions are more similar to the EU average, then the better-off a region is in the distribution, the more it will directly compete with advanced EU imports compared to the poorer regions. Less well-off Greek regions are less likely to face direct substitution effects from EU trade as, they (i) produce lower value-added products and (ii) are more sheltered from any type of trade (Petrakos and Psycharis 2016). Hence, economically more advanced regions will be more vulnerable to increases in EU trade intensity. Poorer regions will, by contrast, be more affected by trade of basic products from the rest of the world.

The use of quantile regressions permits assessing how the impact of EU trade varies with the conditional distribution of regional income (GDP per capita). Our analysis estimates the effect of EU trade at five points of the regional income distribution—for regions at the 0.10, 0.25 0.50, 0.75, and 0.90 income quantiles. By using QR techniques we account for the heterogeneity across regions, allowing the coefficients of our main regressors and of the explanatory variables to differ, thus capturing better the asymmetric effects of EU trade on growth. The QR method can also be thought of as a way to control implicitly for un-modelled growth determinants.

The assumption of parameter homogeneity is neither an empirical nor a theoretical result (Crespo-Cuaresma et al. 2011). From a theoretical point of view, the fact that economic units which are affected by policies, or hit by negative growth shocks, may present different economic dynamics which would “require the specification of a different data-generating process has received attention in the economic growth literature” (Crespo-Cuaresma et al. 2011: 811). This justifies the need for empirical models with parameter heterogeneity.

Empirically, the rationale for using QR is that standard linear regression techniques summarize the average relationship between a set of regressors and the outcome variable based on the conditional mean function *E(y|x).* This provides only a partial view of the relationship. Consequently, analysing the relationship at different points in the conditional distribution of *Y* delivers a more accurate picture of the exact impact of trade at different levels of the regional income distribution. The most important feature of quantile regressions is their ability to estimate quantile-specific effects that describe the impact of covariates not only on the center, but also on the tails of the outcome variable’s distribution. While the central effects, such as the mean effect obtained through conditional mean regression, provide a valuable overview of the impact of a covariate, they fail to describe the full distributional impact unless the variable affects both the central and the tail quantiles in the same way (Chernozhukov and Hansen 2008).

We therefore consider the relationship between the regressors and outcome using the conditional median function where the median is the 50th percentile, or quantile *q*, of the empirical distribution. The quantile q(0;1) is that *y*, which splits the data into proportions *q* below and *1- q* above. QR is also more robust to non-normal errors and outliers.

### Trading with rich and poor countries

Table 1 presents the results for the fixed effects model—answering H_1_—considering first the overall openness to trade in Greece. Their connection between trade openness and GDP per capita growth is negative and significant for the average Greek region, indicating that overall openness has been detrimental for economic growth in Greece. This result is in line with that of Petrakos et al. (2015).

**Table 1:** Fixed effects—overall openness

	(1)	(2)	(3)	(4)
	*Log GDP cap*	*Log GDP cap*	*Log GDP cap*	*Log GDP cap*
Openness	−0.025***	−0.024***	−0.023***	−0.023***
	(0.008)	(0.008)	(0.008)	(0.008)
Pop density (log)	−0.412***	−0.437***	−0.432***	−0.431***
	(0.092)	(0.092)	(0.092)	(0.092)
PIP (log)		−0.021***	−0.020***	−0.021***
		(0.007)	(0.007)	(0.007)
Accessibility			0.057	0.048
			(0.064)	(0.064)
Public share				0.194
				(0.143)
Observations	650	650	650	650
*R*^2^	0.807	0.810	0.810	0.811
Region FE	YES	YES	YES	YES
Year FE	YES	YES	YES	YES

Notes: A constant is included but not reported; all explanatory variables are 1-year lags; Robust standard errors in parentheses ***p < 0.01, **p < 0.05, * *p* < 0.1

Table 2 focuses explicitly on the impact of trade with the generally more developed countries of the EU. As indicated by the negative and statistically significant coefficient, EU trade has not led to greater regional growth in Greece and this connection remains stable across specifications including additional controls. Although EU integration has been sold as a win–win situation for both core and peripheral countries, the industry in EU countries with which Greece trades is far too competitive for the Greek industrial fabric. This has potentially triggered an import substitution effect, leading to a contraction of economic activity in Greece, consequently, affecting GDP growth.

**Table 2:** Fixed effects—EU trade

	(1)	(2)	(3)	(4)	(5)
	*Log GDP cap*	*Log GDP cap*	*Log GDP cap*	*Log GDP cap*	*Log GDP cap*
EU trade	−0.039*	−0.056**	−0.053**	−0.055**	−0.061***
	(0.023)	(0.023)	(0.023)	(0.023)	(0.024)
Pop density (log)	−0.342***	−0.434***	−0.458***	−0.452***	−0.453***
	(0.090)	(0.093)	(0.092)	(0.092)	(0.092)
Openness		−0.029***	−0.028***	−0.027***	−0.028***
		(0.008)	(0.008)	(0.008)	(0.008)
PIP (log)			−0.020***	−0.019***	−0.020***
			(0.007)	(0.007)	(0.007)
Accessibility				0.067	0.056
				(0.149)	(0.141)
Public share					0.252*
					(0.144)
Observations	650	650	650	650	650
*R*^2^	0.804	0.808	0.812	0.812	0.813
Region FE	YES	YES	YES	YES	YES
Year FE	YES	YES	YES	YES	YES

A constant is included but not reported; all explanatory variables are 1-year lags; Robust standard errors in parentheses ***p < 0.01, **p < 0.05, * *p* < 0.1

The coefficient for overall openness remains negative and significant, again highlighting the potential negative effects of trade with more developed countries in the case of Greece. Among the control variables, public investment displays a negative and significant coefficient, which may be attributed to the meager returns of the public investment programme (Rodríguez-Pose et al. 2012), as a possible consequence of the fact that the spatial allocation of investment is surprisingly stable over time and does not follow a logic of efficiency or equity (Monastiriotis and Psycharis 2014). The lack of returns of investment may also reflect 'political inertia' and graft (Monastiriotis and Psycharis 2014:0.451).

The coefficient for population density is negative but not significant. Although population density can trigger agglomeration economies and positive externalities, in the case of Greece this is not translated into higher economic growth. Does this hold in different stages of the economic cycle? We test whether this is the case by stratifying our sample period in two sub-periods: *before* and *during* the crisis (Table 7 in Appendix). The results show that during the crisis large agglomerations in Greece were most affected and more vulnerable (see also Psycharis et al. 2014).

Public expenditure is positive and significant in explaining regional growth while accessibility displays a positive albeit non-significant sign in the baseline models (in contrast to the QR models below).

In Table 3, we present the results of trade with the rest of the world. The positive and significant sign of trade with non-EU countries reveals that trade with generally less developed countries represents a growth stimulus for the average Greek region. This may be a consequence of lower substitution effects and the higher competitiveness of Greek exports in non-EU markets. This result suggests that the EU markets are possibly far too competitive for the internationalization efforts of Greek firms. The main Greek trade partners outside the EU—Turkey, North Africa, China, Balkan countries, Russia—have provided a better opportunity for growth, reflecting Greece’s competitive advantage. Firms in many Greek regions responded to the post-2008 financial crisis by expanding their markets, but this expansion has, more often than not, not included the EU. For reasons related to the fragmented character of their productive base and their specialization, many Greek regions find it difficult to penetrate more advanced markets and have sought trade opportunities in less developed countries outside the EU, where entry requirements may be lower (Petrakos and Psycharis 2016: 146).

**Table 3:** Fixed Effects—ROW (*Non-EU* trade)

	(1)	(2)	(3)	(4)
	*Log GDP cap*	*Log GDP cap*	*Log GDP cap*	*Log GDP cap*
ROW	0.047**	0.043**	0.049**	0.051**
	(0.021)	(0.021)	(0.021)	(0.021)
Pop density (log)	−0.431***	−0.454***	−0.453***	−0.449***
	(0.093)	(0.092)	(0.092)	(0.092)
Openness	−0.029***	−0.028***	−0.029***	−0.028***
	(0.008)	(0.008)	(0.008)	(0.008)
PIP (log)		−0.020***	−0.020***	−0.020***
		(0.007)	(0.007)	(0.007)
Public Share			0.256*	0.244*
			(0.143)	(0.144)
Accessibility				0.058
				(0.064)
Observations	650	650	650	650
*R*^2^	0.808	0.811	0.812	0.813
Region FE	YES	YES	YES	YES
Year FE	YES	YES	YES	YES

A constant is included but not reported; all explanatory variables are 1-year lags; Robust standard errors in parentheses ***p < 0.01, **p < 0.05, * *p* < 0.1

### Trade impact across the Greek regional wealth distribution

But how homogeneous or heterogeneous is the territorial impact of increases in trade across Greek regions? Tables 4 and 5 report the results of the model across the five income quantiles considered, testing H_2_. Table 4 presents the results of the effect of EU trade across regional income quantiles. The impact of EU trade is highly heterogeneous, as revealed by the significance of the variable in the upper quantiles and its insignificance in the lower quantiles. Trade with the richer countries of the EU thus affects to a far larger extent better-off Greek regions. Regions with a medium-income level (0.50), as well as the rich (0.75) and the richest regions (0.90) seem to lose the most from trade with EU partners. By contrast, EU trade does not seem to harm the growth prospects of the poorest (0.10) and other poor regions (0.25) in the lower tails of the income distribution. These regions compete less with EU imports, due to their smaller and less sophisticated tradable sectors. This situation shields them from substitution effects.

**Table 4:** Quantile regressions—EU trade and openness

	(0.10)	(0.25)	(0.50)	(0.75)	(0.90)
	*Log GDP cap*	*Log GDP cap*	*Log GDP cap*	*Log GDP cap*	*Log GDP cap*
EU trade	−0.018	−0.025	−0.047**	−0.069**	−0.055**
	(0.028)	(0.029)	(0.022)	(0.029)	(0.023)
Openness	−0.021**	−0.021**	−0.033***	−0.021**	−0.007
	(0.010)	(0.010)	(0.008)	(0.010)	(0.008)
Pop density (log)	−0.265**	−0.353***	−0.380***	−0.469***	−0.354***
	(0.111)	(0.116)	(0.087)	(0.116)	(0.091)
PIP (log)	−0.020**	−0.018**	−0.013**	−0.014*	−0.010
	(0.008)	(0.008)	(0.006)	(0.008)	(0.006)
Accessibility	0.092	0.062	0.118**	0.237***	0.169***
	(0.077)	(0.080)	(0.060)	(0.080)	(0.063)
Observations	650	650	650	650	650
*R*^2^	0.832	0.824	0.836	0.856	0.884
Region FE	YES	YES	YES	YES	YES
Year FE	YES	YES	YES	YES	YES

A constant is included but not reported; all explanatory variables are 1-year lags; Robust standard errors in parentheses ***p < 0.01, **p < 0.05, * *p* < 0.1

**Table 5:** Quantile Regressions—ROW (*Non-EU* trade)

	(0.10)	(0.25)	(0.50)	(0.75)	(0.90)
	*Log GDP cap*	*Log GDP cap*	*Log GDP cap*	*Log GDP cap*	*Log GDP cap*
ROW	0.033	0.023	0.039**	0.053**	0.018
	(0.025)	(0.027)	(0.020)	(0.027)	(0.021)
Openness	−0.023**	−0.020*	−0.039***	−0.027***	−0.005
	(0.010)	(0.010)	(0.007)	(0.010)	(0.008)
Pop density (log)	−0.259**	−0.332***	−0.386***	−0.467***	−0.320***
	(0.112)	(0.118)	(0.086)	(0.116)	(0.091)
PIP (log)	−0.020**	−0.019**	−0.011*	−0.015*	−0.011*
	(0.008)	(0.008)	(0.006)	(0.008)	(0.006)
Accessibility	0.090	0.070	0.115*	0.217***	0.151**
	(0.079)	(0.083)	(0.061)	(0.082)	(0.064)
Observations	650	650	650	650	650
*R*^2^	0.833	0.824	0.836	0.856	0.884
Region FE	YES	YES	YES	YES	YES
Year FE	YES	YES	YES	YES	YES

A constant is included but not reported; all explanatory variables are 1-year lags; Robust standard errors in parentheses ***p < 0.01, **p < 0.05, * *p* < 0.1

These findings confirm H_1_ : the competitive pressures of trading with more technologically advanced countries are more pronounced in the upper quantiles of the regional income distribution. These regions have experienced economic contraction due to differences in price, cost competitiveness, and quality of producing similar goods and products to those imported from the EU. This is attributed to differences in the economies of scale of the industrial sector in Greece compared to its core trading partners (Germany, Italy, Spain, the Netherlands) and to differences in production efficiency, which intensifies within-industry competition.

The negative growth returns from EU trade (mainly in manufacturing) contrast with the predictions of new trade theory (NTT) and the endogenous growth theory. This partly may reflect the fact that a majority of advanced Greek firms have been incapable of competing and/or branching into global value chains with other firms in the core of the EU, especially as regards backward linkages (i.e., supplying intermediate products). This could have been a positive source of vertical intra-industry trade. Greek firms in general have experienced greater difficulties in this respect than their counterparts in Italy and Spain or in Central and Eastern Europe (Konstantakopoulou 2015). This is likely to have made these regions recipients of cheaper intermediate inputs and of technologically advanced inputs from EU countries, crowding out domestic suppliers, shedding jobs and growth. This trend has also contributed to the large Greek trade deficit (Konstantakopoulou 2015).

An example of the crowding-out of manufacturing activity in the upper quantiles, which coincided with the surge of EU imports into Greece, is the prefecture of Viotia. During Greece’s growth period (from 2000 to 2008)—coinciding with the highest volumes of imports from the EU and the widening of the trade deficit—the upward trend of economic activity included almost all the NUTS III regions/prefectures of the country, with the exception of Viotia (the neighboring region to Attika, where the bulk of the manufacturing activity of Athens, and of Greece as a whole, is located) (Psycharis et al. 2014: 74). Viotia, a region with a similar sectoral structure to the EU imports, has been more exposed to competition pressures and failed to improve its dynamic export capacities.

Table 5 presents the results for regional trade with the ROW across regional income quantiles. The ROW variable returns a positive and significant sign for wealthier regions and an insignificant one for poorer ones. This finding suggests that trade with non-EU countries is beneficial for regions in the top half of the income distribution (between 0.50 and 0.90). While these regions lose out from greater integration with more developed countries, they benefit from trade with less developed ones. Industry in these regions is more competitive in non-EU markets, as it is less exposed to substitution effects from trade with lower-income countries. Hence, the rise in trade with Balkan countries, which intensified in the period 2008–2012, when the Balkans became the most important destination for Greek exports, was beneficial for the main Greek industrial hubs (Tsiapa 2019). As indicated by Tsiapa (2019: 629), “the economic crisis partially suspended the trade co-operation networks between Greece and the EU due to emerging restrictions on financing and credibility, and gave emphasis to more flexible co-operation schemes with the Balkans based on the advantages of geographical proximity and historical ties—while the export share in markets such as Asia and Africa recorded also a notable rise”. This confirms the higher relative competitiveness of Greek exports from the more developed regions to less developed markets.

In contrast, regions below the Greek average in terms of GDP—which are mainly sheltered economies (i.e., relying heavily on the public sector and agricultural subsidies) and have small tradable sectors—did not benefit from the expansion of trade with poorer countries following the outbreak of the financial crisis (Petrakos and Psycharis 2016). During this crisis, Greek regions manifested “an average 13% increase in the level of dependence on sheltered types of activities […] and a decrease of 5% in the participation of the tradable sectors in their GDP” (Petrakos and Psycharis 2016: 146).

As a further robustness check, we replicate the quantile regressions including the public share control variable for both sets of regressions (Appendix Tables 8 and 9). The coefficients of EU trade and ROW trade remain stable in direction and statistical significance, confirming the robustness of the main estimates. Interestingly, the parameter heterogeneity in the QR reveals that public share is positively related to regional growth for the lowest quantile (0.10), which are the sheltered regions, and the richest regions (0.90), where the highest functions of public policy related positions are concentrated.

**Fig. 6:**
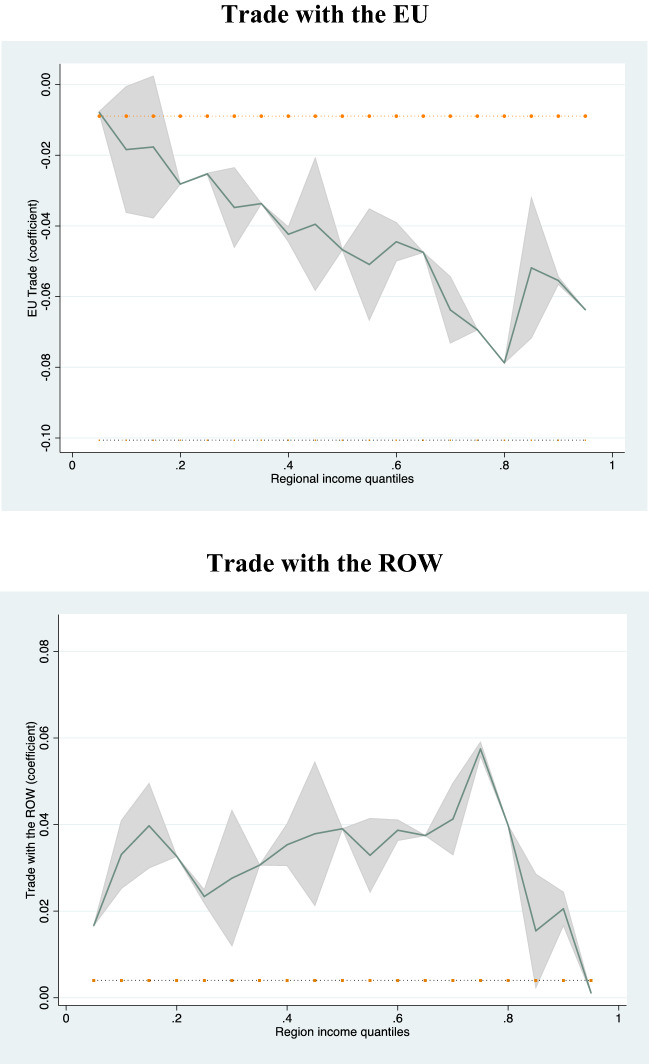
Spatial variation of EU Trade and ROW across regional quantiles

For a visual representation of the heterogeneity of the main regressors, Fig. 6 displays the coefficient of the two key variables across regional quantiles, the dotted line which is set to zero shows that EU trade has a negative effect on regional growth while non-EU trade has a positive effect with fluctuations in magnitude across quantiles. It is evident from the graph that the negative effect of EU trade integration is lower in the lower quantiles and grows as we move up the regional income distribution. By contrast, the positive coefficient of the ROW index increases in size and becomes significant as we go up the regional income distribution (from the 0.50 up to the 0.80), showcasing the positive growth returns—for the more industrialized and more advanced regions—from trade with non-EU countries.

### Endogeneity concerns

We address potential endogeneity concerns by means of system GMM estimators proposed by Blundell and Bond (1998). We also use longer lags of the explanatory variables as instruments of their own current values and to improve efficiency (Roodman 2009). Table 10 (in Appendix) presents the results of the FE regressor and the system GMM estimator, including the standard post estimation tests.

The FE estimator is compared to the GMM estimator with 2–4-year lags. The system GMM estimator confirms the statistical significance and direction of the main regressor. The test statistics for all specifications are in line with expectations. The Arellano-Bond for serial correlation in the first differences of the residual rejects the hypothesis of no first-order serial correlation, while it fails to reject at higher orders, as desired. This allows excluding the presence of residual serial correlation in the original error term. In addition, the Hansen statistics (including the Difference-in-Hansen tests of exogeneity of instrument subsets) are used to test overidentifying restrictions; the Hansen test (Roodman 2009) returns a *p*-value of 0.310, confirming the validity of the selected instruments in the baseline specifications. We can therefore infer that the main regressors are robust both to the inclusion of additional controls and based on the GMM estimation results including the post estimation tests.

## Conclusion

Trade integration has implications for the economic development of the countries involved. In particular, trading with more advanced countries may have different implications than trading with less advanced ones and the impacts may vary according to the level of development of the regions involved in the process. In this study we have explored the impact across the regions of Greece of increases in trade with generally more developed countries—represented by trade with the EU—and trade with less developed countries—represented by the rest of the world.

The results reveal that increases in trade have not been all that beneficial for Greece and, in particular, for some of its regions. There is an overall negative association between increases in trade and economic growth in Greece. Specifically, trade with the more advanced regions of the EU has been connected to lower growth in the most developed regions of Greece, due to dynamics associated with substitution effects. Trade integration with the EU has more pronounced effects in the upper quantiles of the regional income distribution. Richer regions in Greece have been incapable of competing directly with their EU counterparts, due to similarities in the regional sectoral profile, leaving then more vulnerable than less developed regions to trade openness. The benefits that richer regions experience as a consequence of increases in trade with the rest of the world do, however, not compensate for the economic losses of trade integration with the EU.

In line with theoretical and empirical findings of related studies (Krugman and Livas Elizondo 1996; Paluzie 2001; Rodríguez Pose and Gill 2006; Rodríguez-Pose 2012), less developed regions in Greece are also unable to reap the benefits of deeper integration, which is reflected in the absence of positive effects of the EU trade. In more advanced regions substitution effects are at work. Regional industrial growth in Greece’s core has been negatively affected by increases in EU trade (Kallioras and Petrakos 2010; Petrakos et al. 2012). The most advanced regions in Greece have remained not competitive for trade integration with the more advanced partners of the EU. Their relatively weak industrial structures and institutional weaknesses prevent them from reaping the growth-inducing effects of trade resulting from the opening of their markets to more advanced trading partners. Finally, the evidence shows that the median region is the one more likely to lose out from trade integration with richer countries. This has important policy implications for the spatial distribution of future public investment programmes and the Structural Funds allocation objectives that aim to address the negative effects of integration.

The successions of crises that have recently affected Greece—from the global financial crisis to the more recent Covid-19-related pandemic—combined with the absence of counter-veiling policy measures and the low growth returns from trade may trigger a further decline in the growth prospects for more advanced regions. To avoid this, spatially redistributive and regionally sensitive macroeconomic (fiscal) policies and institutional reforms are needed to reverse a vicious cycle that will deprive regions of their real potential. A posteriori, trade integration is not always a panacea for growth. Like with any policy, there are winners and losers from process of trade integration. Complementary policies are needed in combination with a realistic fiscal plans to address the weaknesses related to the structure and geography of Greece and allow its tradable sector to survive and upgrade. These policies could include a customized industrial policy tailored to the needs of the local industry with an emphasis on upgrading the productivity of import competing regions, increase the technological complexity of Greek industry, and allow it to diversify into related sectors. Only by increasing the technological and innovative capacity and the productivity of firms can Greek export competitiveness in higher value-added manufacturing be promoted. Regional policies in Greece should also be designed with the aim of upgrading those import competing regions that have suffered the most from trade integration. This could be done through re-training and on-the-job schemes and by providing financial incentives for the promotion and adoption of innovation by Greek firms. To this end, the public investment programme and the allocation of EU SFs—as well as the Next Generation Recovery fund—need to re-focus on promoting R&D activities and university-industry linkages in high value-added activities with a clear focus on facilitating funding and simplifying the regulatory framework for investment and business creation.

While our research has provided some answers to the problems a country like Greece has experienced when integrating with the trade partners, further research is needed to fine-tune both factors behind this negative impact and provide better solutions to the problem. In particular, further research should focus on industry-specific shocks and the effects on employment and wage outcomes at the regional level, as well as on the compositional nature of employment responses (i.e., low- versus high-skilled labor) in transforming regional labor markets. Moreover, a meaningful task for future empirical investigation can be the replication of this type of analysis in different contexts to assess the validity of the current study and to derive evidence for cross-country and cross-regional comparisons of trading with more and less developed partners.

## Appendix

See Tables 6, 7, 8, 9 and 10.

**Table 6:** List of variables

Variable (code)	Definition	Time & spatial dimension	Source
EU Trade	The ratio of EU imports and exports over total imports and exports of the region	NUTS III 2000–2013	National Statistical Office (ELSTAT)
Log GDP cap	GDP per capita at constant 2005 prices in logs	NUTS III 2000–2013	National Statistical Office (ELSTAT)
Openness	The ratio of total imports and exports over GDP	NUTS III 2000–2013	National Statistical Office (ELSTAT)
Log PopDen	Ratio of population over area (km^2^)	NUTS III 2000–2013	National Statistical Office (ELSTAT)
Log_PIP	The log of the annual per capita expenditures of the Public Investment Programme	NUTS III 2000–2013	Ministry of Economy
Accessibility	Road accessibility is measured as the inverse time-distance weighted population	NUTS II 2000–2013	European Commission DG for Regional and Urban Policy
Public share	GVA of public sector over regional GDP	NUTS II 2000–2013	National Statistical Office (ELSTAT)

**Table 7:** Regression results before and during the crisis

	Pre-crisis: 2000–2008	During crisis: 2009–2013
	*Log GDP/cap*	*Log GDP/cap*
EU Trade	−0.094**	−0.103**
	(0.036)	(0.049)
Openness	−0.055***	−0.038
	(0.014)	(0.032)
Log PopDen	−0.260	−1.301***
	(0.300)	(0.356)
Log PIP	−0.015	0.005
	(0.010)	(0.009)
Observations	400	150
R^2^	0.683	0.835
Region FE	YES	YES
Year FE	YES	YES

All explanatory variables are 1-year lags

Robust standard errors in parentheses ****p* < 0.01, ***p* < 0.05, * *p* < 0.1

**Table 8:** QR regressions—EU Trade (extended versions)

	(0.10)	(0.25)	(0.50)	(0.75)	(0.90)
Variables	*Log GDP cap*	*Log GDP cap*	*Log GDP cap*	*Log GDP cap*	*Log GDP cap*
EU trade	−0.032	−0.029	−0.048**	−0.069**	−0.047**
	(0.029)	(0.030)	(0.023)	(0.029)	(0.023)
Openness	−0.344***	−0.318***	−0.378***	−0.501***	−0.349***
	(0.111)	(0.119)	(0.091)	(0.115)	(0.091)
Pop density (log)	−0.028***	−0.018*	−0.036***	−0.020**	−0.006
	(0.010)	(0.010)	(0.008)	(0.010)	(0.008)
PIP (log)	−0.022***	−0.017**	−0.013**	−0.014*	−0.010
	(0.008)	(0.008)	(0.006)	(0.008)	(0.006)
Accessibility	0.059	0.065	0.107*	0.247***	0.179***
	(0.078)	(0.083)	(0.063)	(0.080)	(0.063)
Public Share	0.385**	0.252	0.054	0.279	0.349**
	(0.174)	(0.185)	(0.142)	(0.180)	(0.142)
Observations	650	650	650	650	650
*R*^2^	0.83	0.82	0.83	0.85	0.88
Region FE	YES	YES	YES	YES	YES
Year FE	YES	YES	YES	YES	YES

Robust standard errors in parentheses ****p* < 0.01, ***p* < 0.05, * *p* < 0.1

**Table 9:** QR regressions—*non*EU Trade (extended versions)

	(0.10)	(0.25)	(0.50)	(0.75)	(0.90)
Variables	*Log GDP cap*	*Log GDP cap*	*Log GDP cap*	*Log GDP cap*	*Log GDP cap*
ROW	0.041	0.026	0.041**	0.047*	0.021
	(0.026)	(0.028)	(0.021)	(0.026)	(0.021)
Openness	−0.318***	−0.307**	−0.389***	−0.490***	−0.340***
	(0.111)	(0.120)	(0.090)	(0.115)	(0.092)
Pop density (log)	−0.032***	−0.019*	−0.040***	−0.026**	−0.005
	(0.010)	(0.010)	(0.008)	(0.010)	(0.008)
PIP (log)	−0.024***	−0.018**	−0.011*	−0.014*	−0.010
	(0.008)	(0.009)	(0.006)	(0.008)	(0.007)
Accessibility	0.052	0.052	0.106*	0.226***	0.157**
	(0.077)	(0.083)	(0.063)	(0.080)	(0.064)
Public Share	0.444**	0.250	0.018	0.269	0.375***
	(0.173)	(0.187)	(0.141)	(0.179)	(0.143)
Observations	650	650	650	650	650
*R*^2^	0.88	0.83	0.88	0.85	0.83
Region FE	YES	YES	YES	YES	YES
Year FE	YES	YES	YES	YES	YES

Robust standard errors in parentheses ****p* < 0.01, ***p* < 0.05, * *p* < 0.1

**Table 10:** Dynamic panel-data estimation, system GMM

	FE	System GMM (2 -4 instruments)
Variables	*(1)*	*(2)*
EU Trade	−0.053**	−0.201**
	(0.023)	(0.076)
Controls Observations	YES 650	YES 650
Year FE	YES	YES
Region FE	YES	NO
Hansen statistic	–	5.96
*p*-value		0.310
AR(1) statistic	–	−4.72
*p*-value		0.000
AR(2) statistic	Variables	−0.35
*p*-value		0.725

Dependent variable is the log GDP per cap.; robust standard errors in parentheses clustered at the region level; ****p* < 0.01, ***p* < *0.05, * p* < 0.1

## Footnotes

1 'Sheltered' regions are those economies that rely heavily on agricultural subsidies and the public sector with a low integration in the global economy (Petrakos and Psycharis 2016). As stressed in Fratesi and Rodríguez-Pose (2016) ‘sheltered’ economies are those “more impervious to changes in the business cycle; with an economic structure less capable of taking advantage of high growth periods” (Rodríguez-Pose and Fratesi 2007: 624).

2 With the exception of the south of Mexico, which ontinues to lag behind.

3 These include two sectors, two regions, and the emphasis on the relocation of manufacturing activity due to free trade. The main difference between Krugman and Livas Elizondo's (1996) and Paluzie’s models is that the centrifugal force is the congestion cost in the former model, while in the latter it is the agricultural population, which is tied to the land.
